# Identification and Characterization of Jasmonic Acid Methyltransferase Involved in the Formation of Floral Methyl Jasmonate in *Hedychium coronarium*

**DOI:** 10.3390/plants13010008

**Published:** 2023-12-19

**Authors:** Yuechong Yue, Xiaohong Zhang, Lan Wang, Jieling He, Shengnan Yang, Xinyue Li, Yunyi Yu, Rangcai Yu, Yanping Fan

**Affiliations:** 1 The Research Center for Ornamental Plants, College of Forestry and Landscape Architecture, South China Agricultural University, Guangzhou 510642, China; ycyue@scau.edu.cn (Y.Y.); zhangxiaohong834@163.com (X.Z.); wanglan@stu.scau.edu.cn (L.W.); hejieling@cqu.edu.cn (J.H.); lixinyue1203@126.com (X.L.); hyphen950@163.com (Y.Y.); 2 Guangdong Key Laboratory for Innovative Development and Utilization of Forest Plant Germplasm, South China Agricultural University, Guangzhou 510642, China; 3 College of Life Sciences, South China Agricultural University, Guangzhou 510642, China; rcyu@scau.edu.cn

**Keywords:** *Hedychium coronarium*, JMT, SABATH methyltransferase, methyl jasmonate, floral volatiles

## Abstract

*Hedychium coronarium* is a popular ornamental flower in tropical and subtropical areas due to its elegant appearance and inviting fragrance. Methyl jasmonate (MeJA) is one of the volatile compounds in the blooming flowers of *H. coronarium*. However, the molecular mechanism underlying floral MeJA formation is still unclear in *H. coronarium*. In this study, a total of 12 SABATH family genes were identified in the genome of *H. coronarium*, and their encoded proteins range from 366 to 387 amino acids. Phylogenetic analysis revealed seven clades in the SABATH family and a JMT ortholog clade, including two HcSABATH members. Combined with expression profiling of HcSABATH members, *HcJMT1* was identified as the top candidate gene for floral MeJA biosynthesis. In vitro enzyme assays showed that HcJMT1 can catalyze the production of MeJA from jasmonic acid. Gene expression analysis indicated that *HcJMT1* exhibited the highest expression in the labella and lateral petals, the major sites of MeJA emission. During flower development, the two MeJA isomers, major isomers in the products of the HcJMT1 protein, were released after anthesis, in which stage *HcJMT1* displayed high expression. Our results indicated that HcJMT1 is involved in the formation of floral MeJA in *H. coronarium*.

## 1. Introduction

Floral volatiles are complex mixtures of many low-molecular-weight and low-boiling-point lipophilic molecules emitted by flowers [1]. Their main function is to serve as signaling molecules to attract pollinators, enabling them to accurately identify the plant species and location, thereby enhancing pollination efficiency [2]. Similarly to volatiles released by other plant organs, floral volatiles can also directly repel some pathogens, parasites and herbivores to protect delicate floral tissues or mediate the interaction between plants to induce defensive responses [3,4]. In addition, floral volatiles can confer resistance to the abiotic stresses of the surrounding environment, such as temperature and oxidative stress [5]. From a human perspective, floral volatiles are one of the important aesthetic and commercial traits for ornamental plants, which have gained increasing attention in recent years [6,7]. Moreover, floral volatile compounds are widely applied in cosmetics, perfumes, flavorings, and medicinal applications [1]. To date, more than 1700 floral volatiles have been identified in over 1000 plant species, which can be mainly categorized into terpenoids, phenylpropanoids/benzenoids, and fatty acid derivatives [1,8]. Compared to the first two categories, fewer studies have focused on the biosynthesis of fatty acid derivatives in flowers, such as floral methyl jasmonate (MeJA).

MeJA is a common component of floral volatiles and is initially identified from the flowers of *Jasminum grandiflorum* [9]. It often emerges in the floral scent profiles of ornamental plants, such as *Cymbidium ensifolium* [10], *C. faberi* [11], *Paeonia ostia* [12], and *Hedychium coronarium* [13]. In *C. ensifolium*, MeJA accounts for the largest proportion of floral volatiles, and its emission is perianth-specific and floral development-regulated [10,14]. Moreover, the emission occurs during the daytime, which is correlated with the foraging activity of its potential pollinator honeybee, indicating the possible attraction function of MeJA relative to pollinators [14]. MeJA is the methylated product of jasmonic acid, whose biosynthesis is initiated from α-linolenic acid, followed by serial catalyzed steps [15]. The conversion of jasmonic acid into MeJA is mediated by jasmonic acid methyltransferase (JMT) in the SABATH methyltransferase family [16,17]. The JMTs responsible for MeJA biosynthesis have been characterized in several plants [18,19,20,21]. However, the JMTs that contribute to MeJA production in flowers were only characterized in *C. ensifolium* and *C. faberi* [11,14]. Functional characterization in vitro showed that CeJMT can synthesize MeJA from jasmonic acid and S-adenosyl-L-methionine (SAM) substrates. The expression pattern of the *CeJMT* gene in flowers comprises flower development and diurnal rhythms, which coincide with MeJA emissions, suggesting its involvement in the metabolic regulation of floral MeJA emissions in *C. ensifolium* [14].

SABATH methyltransferases are an important class of plant methyltransferases that play significant roles in plant growth, development, defense response, and pollination biology [22,23]. Distinct from other types of plant methyltransferases, SABATH members possess unique three-dimensional structures and amino acid sequences, and they catalyze the methylation of the carboxyl group or the nitrogen atom of small molecules [24]. Many volatile methyl esters, such as MeJA, methyl salicylate, methyl benzoate, methyl cinnamate, and methyl anthranilate, are synthesized by the action of SABATH methyltransferases [24,25,26]. The first member reported in the SABATH family is salicylic acid methyltransferase (SAMT) from *Clarkia breweri*, which catalyzes the formation of the floral scent compound methyl salicylate [27]. Subsequently, benzoic acid methyltransferase (BAMT) was characterized in snapdragon [28], and theobromine synthase was investigated in the tea plant [29]. Based on the names of the first three identified members, the family was named the SABATH methyltransferase family [22]. The SABATH family is widely present in higher plants, with the moss *Physcomitrella patens* and gymnosperm *Picea abies* genomes containing four and ten SABATH family genes, respectively [30,31]. There are 20~32 SABATH members in dicots, such as 20 members in tomato, 24 members in Arabidopsis, 28 members in poplar, and 32 members in *Camellia sinensis*, while 41 SABATH family members are observed in the monocot rice [22,32,33,34,35].

*H. coronarium*, commonly known as the white ginger lily, is a popular ornamental flower in the Zingiberaceae family, which is often applied as a cut flower and landscape plant in tropical and subtropical regions [36]. When blooming, its flowers release up to 28 volatile compounds, including terpenoids linalool and (E)-β-Ocimene, benzenoids/phenylpropanoids methyl benzoate, and the fatty acid derivative MeJA [13]. Several key genes contributing to the biosynthesis of floral terpenoids and benzenoids/phenylpropanoids have been identified and functionally characterized [36,37,38]. For example, two SABATH family members, HcBSMT1 and HcBSMT2, have been demonstrated to be benzoic acid/salicylic acid methyltransferases (BSMTs) in vitro and in planta. Furthermore, enzyme kinetics and gene expression analysis indicated that HcBSMT2 plays a dominant role in the formation of the floral volatile compound methyl benzoate [36]. However, the molecular mechanism of floral MeJA production in *H. coronarium* is still unclear.

In this study, we identified the SABATH family members in the genome of *H. coronarium* and analyzed their gene structures and chromosome distributions. Using the phylogenetic analysis and expression profiling of HcSABATH members, *HcJMT1* was identified as the candidate gene for the formation of floral MeJA and then functionally characterized. Finally, the spatial and temporal expression of *HcJMT1* and the emission of MeJA during the flower development were investigated and discussed.

## 2. Results

### 2.1. Identification of SABATH Gene Family Members in H. coronarium

**Figure 1:**
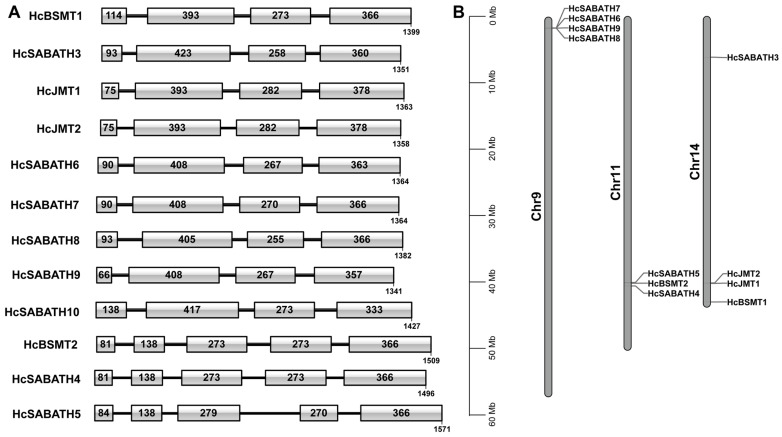
Gene structure and chromosome distribution of SABATH members in *H. coronarium*. (**A**) Exon–intron structure of *HcSABATHs*. The frames and bold lines represent the position of the exon and intron, respectively. The numerals in the frames indicate the length of the exon. The gene length is labeled under the last frame of each gene; (**B**) The distribution of *HcSABATHs* on *H. coronarium* chromosomes.

Given that JMT belongs to the SABATH methyltransferase family, we first analyzed the SABATH family in the genome of *H. coronarium*. Using local blast searches and conserved motif verification, a total of 12 complete SABATH family genes were identified in the genome of *H. coronarium*, designated from *HcSABATH1* to *HcSABATH12* (Table 1). Among them, *HcSABATH1* and *HcSABATH2* represent the functionally characterized BSMT genes *HcBSMT1* and *HcBSMT2*, respectively [36], and *HcSABATH11* and *HcSABATH12* are subsequently renamed as *HcJMT1* and *HcJMT2* for their close evolutionary relationship with JMT genes (Table 1). The gene lengths of *HcSABATHs* range from 1341 to 1571 bp, which contain three or four introns (Figure 1A). The open reading frames (ORFs) of *HcSABATHs* range from 1341 to 1427 bp, encoding 366~387 amino acids. The estimated molecular weights of HcSABATHs are between 38,197.02 and 43,220.25 Da, and the estimated pI values range from 4.96 to 6.11 (Table 1 and Figure 1A).

Except for *HcSABATH10* with an ambiguous chromosomal location, the other 11 *HcSABATH* genes were located on 3 chromosomes of *H. coronarium* and exhibited uneven distribution across the chromosomes. Among them, *HcSABATH6~9*, *HcBSMT2* and *HcSABATH4/5*, as well as *HcJMT1/2*, were tandemly arranged on chromosomes Chr9, Chr11, and Chr14, respectively (Figure 1B). Moreover, the nucleotide sequence similarity between these tandem genes is relatively high, indicating that they might be generated via gene duplication. Gene structure analyses showed that *HcSABATH* genes possess analogous exon–intron structures and similar intron phases (Figure 1A). *HcBSMT2* and *HcSABATH4/5* encompass five exons and four introns each, while the remaining *HcSABATH* genes contain four exons and three introns (Figure 1A), implying that an intron insertion event might occur in the second exon of the ancestral gene of *HcBSMT2* and *HcSABATH4/5*.

### 2.2. Phylogenetic and Expression Profiling Analyses Revealed HcJMT1 as a Candidate for Floral MeJA Formation

**Figure 2:**
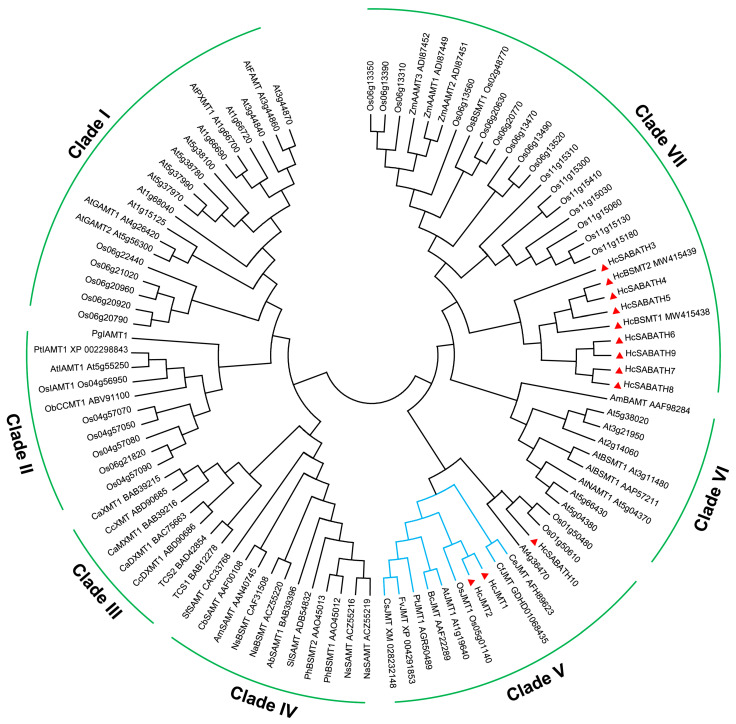
Phylogenetic analysis of HcSABATHs with Arabidopsis and rice SABATHs and other functionally characterized SABATHs. The SABATH members in *H. coronarium* are labeled with red triangles. The JMT clade is indicated in blue. Ab, *Atropa belladonna*; Al, *Arabidopsis lyrata*; Am, *Antirrhinum majus*; At, *Arabidopsis thaliana*; Bc, *Brassica campestris*; Ca, *Coffea arabica*; Cb, *Clarkia breweri*; Cc, *Coffea canephora*; Ce, *Cymbidium ensifolium*; Cf, *C. faberi*; Cs, *Camellia sinensis*; Fv, *Fragaria vesca*; Na, *Nicotiana alata*; Ns, *N. suaveolens*; Ob, *Ocimum basilicum*; Os, *Oryza sativa*; Pg, *Picea glauca*; Ph, *Petunia hybrid*; Pt, *Populus trichocarpa*; Sf, *Stephanotis floribunda*; Sl, *Solanum lycopersium*; Zm, *Zea mays*.

To identify the JMT orthologs in *H. coronarium*, phylogenetic analysis was conducted using SABATH family members in Arabidopsis and rice, as well as functionally characterized plant SABATHs. The phylogenetic tree revealed seven clades, designated from Clade I to VII, and HcSABATHs fell into Clade V and Clade VII (Figure 2). Clade VII is a monocot-specific SABATH subfamily, which is composed of HcBSMT1/2 and HcSABATH3-9, as well as SABATHs from rice and maize (Figure 2). Nine HcSABATHs within Clade VII form a monophyletic branch, which diverges from SABATHs in rice and maize (Figure 2), suggesting that their common ancestor existed before the separation of Zingiberales and Poales lineages and multiple gene-duplication events occurred in Clade VII SABATHs during the evolution of Zingiberales and Poales lineages. As displayed in Figure 2, Clade V comprises angiosperm JMTs and four SABATHs with unknown functions. The angiosperm JMT forms a well-defined monophyletic branch, indicating the stronger conservation of JMT in angiosperms. Two HcSABATHs were identified in the group of JMT orthologs, named HcJMT1 and HcJMT2, which have a closer evolutionary relationship with OsJMT (Figure 2).

**Figure 3:**
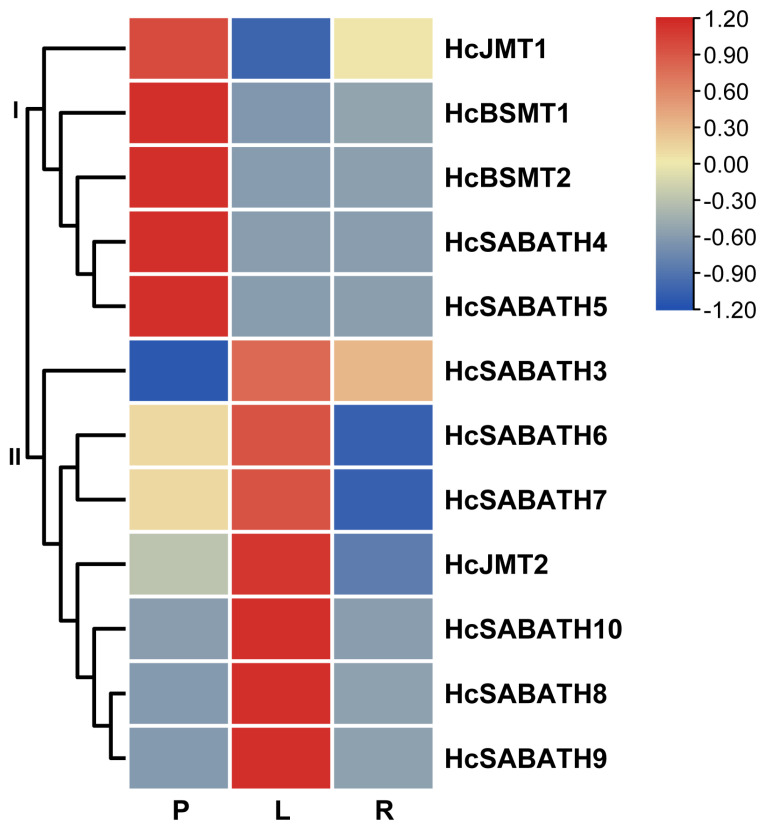
Expression profiles of *HcSABATH* genes in different tissues based on transcriptomic data. P, petals; L, leaves; R, rhizomes.

To screen the highly expressed *HcJMT* in the flowers of *H. coronarium*, the expression profiles of *HcSABATH* genes in different tissues were analyzed using transcriptomic data. The results show that the expression of *HcSABATH* genes exhibited tissue specificity, and their expression profiles clustered into two major categories (Figure 3). The genes in category I displayed the highest expression in petals, including *HcJMT1*, *HcBSMT1/2*, and *HcSABATH4/5*, while category II genes displayed the highest expression in leaves, including *HcJMT2*, *HcSABATH3*, and *HcSABATH6-10* (Figure 3). Thus, taking the results together, *HcJMT1* is the top candidate gene for floral MeJA formation in *H. coronarium*.

### 2.3. Cloning and Sequence Analysis of HcJMT1

**Figure 4:**
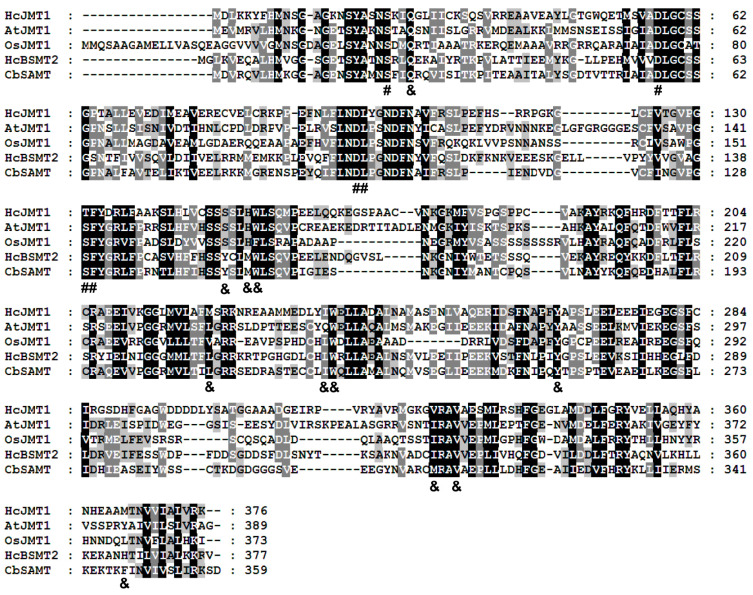
Alignment of amino acid sequences of HcJMT1 with other plant functionally characterized SABATHs. Amino acid residues shaded in black, gray, and light gray represent 100, 80, and 60% conserved identity, respectively. Dashes indicate gaps inserted for optimal alignment. Residues with “#” below indicate SAM binding sites. The amino acids with “&” below indicate substrate binding sites.

To verify the function of *HcJMT1*, we cloned the full-length cDNA sequence of *HcJMT1*. Sequencing analysis revealed that *HcJMT1* has a putative ORF of 1131 bp, encoding 376 amino acid residues with a molecular mass of 41,686.44 Da and a PI value of 5.42 (Table 1 and Figure 4). The alignment of amino acid sequences showed that HcJMT1 shares 40% identity with Arabidopsis AtJMT1 and rice OsJMT1, while it shows a sequence identity of 37% with respect to HcBSMT2 (Figure 4), which exhibits methyltransferase activity towards benzoic acid and salicylic acid in *H. coronarium* [36]. Multiple sequence alignment revealed that almost all residues for SAM binding in HcJMT1 are identical with other functionally characterized SABATHs, indicating the methyltransferase activity of HcJMT1 (Figure 4). Moreover, HcJMT1 contains similar substrate binding sites with respect to Arabidopsis and rice JMT (Figure 4), suggesting the possible JMT activity of HcJMT1. In addition, two residues (Ser-149 and His-152 in HcJMT1) for substrate binding in three JMTs are distinct from the residues (Tyr and Met) in HcBSMT2 and CbSAMT (Figure 4), possibly suggesting different substrate specificities.

### 2.4. HcJMT1 Is a Jasmonic Acid Methyltransferase

**Figure 5:**
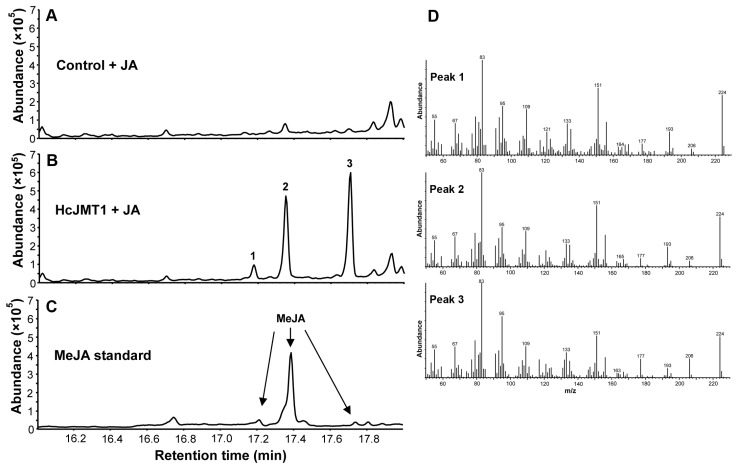
Characterization of HcJMT1 in vitro. (**A**) Total ion chromatogram of the products generated by incubating the crude protein extracts of the empty vector control with jasmonic acid (JA); (**B**) Total ion chromatogram of reaction products formed by recombinant HcJMT1 with the substrate JA; (**C**) Total ion chromatogram of the methyl jasmonate (MeJA) authentic standard. The chromatogram peaks indicated by arrows are different stereoisomers of MeJA; (**D**) Mass spectra of the reaction products in (**B**).

To characterize the function of HcJMT1, the recombinant protein was expressed in *Escherichia coli*, and its methyltransferase activity was tested using jasmonic acid as the substrate in vitro. The result showed that HcJMT1 catalyzed the reaction of jasmonic acid and SAM to produce three chromatographic peaks of products, while no methylated product was detected in the empty vector control (Figure 5A,B). By comparing the retention times and mass spectra of MeJA standards, it was found that the three products are three stereoisomers of MeJA (Figure 5). Similar results were also observed in the catalytic reaction of rice OsJMT1, which could synthesize three stereoisomers of MeJA from jasmonic acid [16]. Thus, HcJMT1 is a jasmonic acid methyltransferase.

To investigate the substrate specificity of HcJMT1, we analyzed the catalytic activity of HcJMT1 towards nine carboxylic acid substrates, dihydrojasmonic acid, salicylic acid, benzoic acid, 3-hydroxybenzoic acid, 2,3-dihydroxybenzoic acid, cinnamic acid, o-anisic acid, o-coumaric acid, and nicotinic acid. The results showed that HcJMT1 only exhibited catalytic activity toward dihydrojasmonic acid, a structural analogue of jasmonic acid (Table 2), indicating that HcJMT1 has strict substrate specificity.

### 2.5. Expression Analysis of HcJMT1

**Figure 6:**
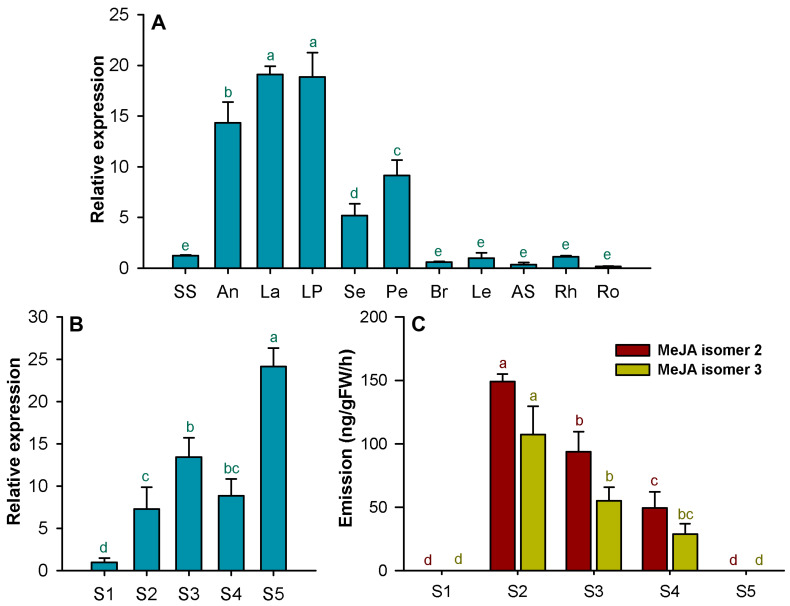
Expression of *HcJMT1* and emission of MeJA in *H. coronarium*. (**A**) Expression analysis of the *HcJMT1* gene in different tissues. SS, styles and stigmas; A, anthers; La, labella; LP, lateral petals; Se, sepals; Pe, pedicels; Br, bracts; Le, leaves; AS, aerial stems; Rh, rhizomes; Ro, roots; (**B**) Expression analysis of the *HcJMT1* gene in petals at different floral developmental stages; (**C**) Emission of floral MeJA isomers at different flower developmental stages. S1, flower bud stage; S2, full-opening stage; S3, 8 h after full-opening; S4, 16 h after full-opening; S5, senescence stage. Different lowercase letters labelled on bars indicate statistically significant differences at the level of *p* < 0.05.

To explore the role of *HcJMT1* in the biosynthesis of floral MeJA in *H. coronarium*, the spatial and temporal expression levels of *HcJMT1* were analyzed via real-time PCR. The results showed that the *HcJMT1* gene was the most highly expressed in petals, including labella and lateral petals, which are the major sites of MeJA emission. Moreover, the *HcJMT1* gene was also expressed in anthers, sepals, and pedicels and only exhibited weak expression levels in styles and stigmas, bracts, leaves, aerial stems, rhizomes, and roots (Figure 6A). During flower development, the expression of *HcJMT1* was low in petals at the flower bud stage (S1); it increased gradually after flowering, peaking at the S3 stage; then, it decreased at the S4 stage; finally, it increased again during the senescence stage (Figure 6B). Interestingly, we detected two MeJA isomers in the flowers of *H. coronarium*, isomer 2 and isomer 3, which are the main products of the HcJMT1 protein (Figure 5 and Figure 6C). The emission patterns of MeJA isomer 2 and isomer 3 in flowers were coincident. Their emissions were not observed at the bud stage (S1), and the release levels were the highest at the full-opening stage and then decreased gradually (Figure 6C).

## 3. Discussion

### 3.1. Evolution of the SABATH Methyltransferase Family in H. coronarium

The SABATH family is a plant-specific group of methyltransferases that are widely present in higher plants [39]. Here, we identified 12 complete SABATH family genes in the genome of the monocot *H. coronarium* (Table 1). Phylogenetic analysis of HcSABATHs with Arabidopsis and rice SABATHs and other functionally characterized SABATHs revealed that the SABATH family can be divided into seven subfamilies (Figure 2). Clade II is mainly composed of plant indole-3-acetic acid methyltransferases (IAMTs), which regulate auxin homeostasis and plant development [40]. Previous studies based on phylogenetic and crystal structure analyses have shown that IAMT is an evolutionarily ancient member of the SABATH family, while other SABATH methyltransferases, such as SAMTs, BAMTs, and JMTs, might evolve from IAMT via gene duplication and functional divergence [32]. Clade I contains Arabidopsis gibberellin methyltransferases (GAMTs), fatty acid methyltransferase (FAMT), and 1,7-dimethylxanthine methyltransferase (PXMT). Recent studies have shown that GAMT orthologs are present in mosses, ferns, lycophytes, gymnosperms, and angiosperms, forming a distinct evolutionary branch [39], while IAMT has only been found in seed plants, indicating that GAMT may be a more ancient member of the SABATH family. In *H. coronarium*, we did not identify any members of Clade I and Clade II (Figure 2), which might result from the loss of members in these clades during the *Hedychium* evolution. A similar scenario has also been observed in other plants, where out of 248 angiosperms, only 69 could be identified with GAMT orthologs [39].

The members in Clade III are primarily involved in the biosynthesis of caffeine, catalyzing the methylation of the nitrogen atom of substrates (Figure 2). Clade IV and VI are dicot-specific SABATH subfamilies, and their members mainly catalyze the formation of volatile methyl esters. Clade VII is a monocot-specific SABATH subfamily, including functionally characterized BSMTs and anthranilic acid methyltransferases (AAMTs). Within Clade VII, nine HcSABATHs and twenty Poales SABATHs cluster in two separate evolutionary branches, suggesting that their common ancestor existed before the divergence of Zingiberales and Poales lineages, and the gene family expansion of this subfamily occurred during the evolution of Zingiberales and Poales lineages. Analyses of the chromosome distribution, sequence similarity, and gene structure of *HcSABATHs* revealed that tandem gene duplication might be the reason for SABATH gene family expansion in *H. coronarium* (Figure 1). Among 41 SABATH genes in the rice genome, 22 are situated in 6 gene clusters formed by tandem repeat genes [32]. This phenomenon was also observed in other plants [33,34,35], indicating that tandem gene duplication is an important factor that drives the expansion of the SABATH gene family in plants.

Angiosperm JMTs could form an apparent monophyletic branch in Clade V (Figure 2). Compared to SAMTs or BSMTs in angiosperms, JMTs appear to undergo stronger selective pressure, which is possibly due to their important role in plant growth, development, and defense response, leading to their higher conservation in angiosperms [16]. This observation suggests that homologous gene retrieval and phylogenetic analysis can help identify JMT homologs in angiosperms. Phylogenetic analysis showed that JMTs in monocots are distinctly divergent from those in dicots, suggesting that the ancestral JMT seems to have existed before the split of monocots and dicots. In *H. coronarium*, two HcJMTs fell into the JMT clade (Figure 2), and only *HcJMT1* exhibited gene expression in flowers (Figure 3), implying its potential role in the biosynthesis of floral MeJA.

### 3.2. Involvement of HcJMT1 in the Biosynthesis of Floral MeJA

MeJA is one of the floral volatile compounds in *H. coronarium* [13]. However, its biosynthesis mechanism remains unclear. JMT represents the key enzyme for MeJA biosynthesis, which catalyzes the production of MeJA from jasmonic acid [16,17]. To date, only two JMTs that contribute to floral MeJA production have been characterized [11,14]. In *C. ensifolium*, the *CeJMT* gene displayed floral developmental and diurnal rhythmic expression, and its recombinant protein could catalyze jasmonic acid to form MeJA [14]. In this study, via phylogenetic analysis and expression profiling of HcSABATH members, we identified a candidate JMT gene, *HcJMT1*, which exhibited the highest expression in petals, the major sites of MeJA emission (Figure 6A). Functional characterization in vitro revealed that HcJMT1 can catalyze jasmonic acid to produce three isomers of MeJA (Figure 5). Meanwhile, in the flowers of *H. coronarium*, we also detected two isomers of MeJA, isomer 2 and isomer 3, which are two major isomers in the products of the HcJMT1 protein (Figure 5 and Figure 6C). Furthermore, the two MeJA isomers shared an identical emission pattern and were released after anthesis (S2~S3), in which stage the *HcJMT1* gene also showed high expression levels (Figure 6B,C). Collectively, we conclude that HcJMT1 is involved in the formation of floral MeJA in *H. coronarium*. We also observed that the *HcJMT1* gene was highly expressed in petals at the senescence stage, and it was expressed moderately in anthers and pedicels. Nevertheless, no MeJA emission was detected in these samples (Figure 6). This observation might result from the shortage of jasmonic acid supply in these samples. Thus, the amount of substrate jasmonic acid in *H. coronarium* should be determined in the future.

The enzymes in the SABATH family often have wide substrate specificity, and more than 59 plant metabolites can be used as potential substrates for SABATH methyltransferases [41], whereas HcJMT1 only exhibited catalytic activity towards jasmonic acid and its structural analogue, dihydrojasmonic acid (Table 2). Similar catalytic activities were also observed for JMTs in Arabidopsis and *C. ensifolium* [14,17], indicating the strict substrate specificity of JMTs. Multiple sequence alignment revealed that two amino acids (equivalent to Ser-149 and His-152 in HcJMT1) for substrate binding in HcJMT1, AtJMT1, and OsJMT1 are conserved, which are different from those in HcBSMT2 and CbSAMT (Tyr and Met) (Figure 4). When the two amino acids in CbSAMT mutated into serine and histidine, the mutated CbSAMT exhibited catalytic activity towards jasmonic acid [42]. In contrast, substituting the serine residue with tyrosine in poplar PtJMT1 led to a 97% decrease in its catalytic activity towards jasmonic acid [18]. These results indicate the significant role of the two amino acids in JMTs with respect to substrate specificity.

### 3.3. Possible Roles of Floral MeJA in H. coronarium

MeJA is one of the important floral scent compounds in ornamental plants. For example, MeJA accounts for the largest proportion of floral volatiles in *C. ensifolium*, and it is a major contributor to the fragrance of the flowers of *C. ensifolium*, which might function in attracting pollinators [14]. In contrast, MeJA is a minor constituent in the floral scent profile of *H. coronarium* [13], suggesting its limited contribution to floral fragrance and pollinator attraction. Investigations reveal that jasmonates, which include jasmonic acid and its amino acid conjugates, and MeJA, play central roles in floral development and maturation [43]. The overexpression of *AtJMT* and the silencing of the *NaMJE* gene in *Nicotiana attenuate* redirects JA towards MeJA, resulting in abnormal expansion of corolla limbs, style shortening, and impaired nectary glands development [44]. Similarly, the heterologous expression of *AtJMT* in rice increased MeJA levels by 6-fold in young panicles and inhibited spikelet development, reducing grain yields [45]. These results indicate that the increased levels of MeJA and decreased levels of JA mediated by JMT might impair flower development and opening. In the flowers of *H. coronarium*, *HcJMT1* showed lower expression in the flower bud stage (S1), while it expressed highly after flowering (S2~S4), which resulted in the emission of MeJA (Figure 6B,C). Thus, *HcJMT1* might coordinate jasmonic acid homeostasis in the process of flower development and opening in *H. coronarium*. In addition, *HcJMT1* displayed the highest expression at the senescence stage (S5), while the emission of MeJA was undetectable at this stage, possibly suggesting a different function of the *HcJMT1* gene besides MeJA synthesis activity.

## 4. Materials and Methods

### 4.1. Plant Materials

*H. coronarium* was cultivated in a horticulture chamber in South China Agricultural University (Guangzhou, China) under natural light. For gene expression analysis, the *H. coronarium* plant at anthesis was divided into 11 tissues: styles and stigmas (SS), anthers (An), labella (La), lateral petals (LP), sepals (Se), pedicels (Pe), bracts (Br), leaves (Le), aerial stems (AS), rhizomes (R), and roots (Ro). The petals at five different floral developmental stages were collected with three biological replicates. All samples were collected in October with ~12 h day length, and they were immediately frozen in liquid nitrogen and stored at −80 °C.

### 4.2. Genome-Wide Identification of SABATH Genes in H. coronarium

To identify the SABATH members in *H. coronarium*, the functionally characterized Arabidopsis SABATHs, including AtJMT1 [17], AtBSMT1 [46], and AtIAMT1 [32], were used as query sequences for the local blast search against the genome of *H. coronarium* with an E-value threshold of 10^−5^. The candidate genes were confirmed using the conserved domain of SABATH methyltransferases (pfam03492) by NCBI CD-search and Pfam 36.0 software [47,48]. The exon–intron structure of *HcSABATHs* was visualized using the IBS 1.0.2 software based on the genome annotation of *H. coronarium* [49]. The distribution of *HcSABATHs* on *H. coronarium* chromosomes was analyzed and visualized using TBtools 1.0 software [50].

### 4.3. Phylogenetic Analysis and Expression Profiling of HcSABATH Genes

Phylogenetic analysis was conducted using SABATH family members identified in Arabidopsis and rice genome [22,32], as well as other functionally characterized SABATHs. Multiple sequence alignment of protein sequences was performed using ClustalW, and an unrooted tree was constructed with MEGA6 using the neighbor-joining method. Evolutionary distances were computed using Poisson model with 1000 bootstrap replicates. The expression patterns of *HcSABATH* genes in different tissues were analyzed based on our transcriptome data [51] and visualized using TBtools 1.0 software [50].

### 4.4. Isolation of the HcJMT1 Gene

The full-length cDNA sequence of *HcJMT1* was amplified using LA Taq DNA polymerase (TaKaRa, Dalian, China) with the gene-specific primers listed in Table S1. The PCR product was ligated into the pMD19-T vector (TaKaRa, Dalian, China) and confirmed by Sanger sequencing. Amino acid sequence alignment was performed using ClustalX and shaded with GeneDoc. The amino acid residues that represented SAM and substrate binding sites were identified from the crystal structures of CbSAMT and AtIAMT1 [32,42]. The *HcJMT1* sequence has been deposited in the GenBank database under accession number OK181913.

### 4.5. Bacterial Expression and Purification of the HcJMT1 Recombinant Protein

The coding region of *HcJMT1* was amplified using LA Taq DNA polymerase (TaKaRa, Dalian, China) with the primers listed in Table S1 and then ligated into the pET-28a vector (Novagen, Darmstadt, Germany) through the cloning sites *Sal*I and *Not*I using T4 DNA ligase (TaKaRa, Dalian, China). The recombinant vector verified via sequencing was transformed into the *E. coli* Rosetta (DE3) strain (Invitrogen, Carlsbad, CA, USA) for recombinant protein induction and purification, as described previously [36,37]. Briefly, the recombinant protein was induced with IPTG for 16 h at 16 °C and purified using Ni-NTA His·Bind Resins (Novagen, Darmstadt, Germany) following the manufacturer’s introduction. The pET28a empty vector was used as a negative control.

### 4.6. In Vitro Enzyme Assays of HcJMT1

To determine the activity of HcJMT1, enzyme assays were conducted in a sealed glass vial with a total volume of 1 mL consisting of 50 mM of Tris-HCl (pH 7.0), 2 mM of SAM, 2 mM of substrate (Table 2), and 100 μL of partial purified HcJMT1 protein. After incubation at 25 °C for 1 h, a solid-phase microextraction (SPME) fiber (Supelco, Bellefonte, PA, USA) was inserted into the reaction vial to adsorb volatile products for 30 min. Then, adsorbed volatiles were analyzed via a gas chromatography-mass spectrometry (GC-MS) system with Agilent 7890A GC and Agilent 5975C MSD (Agilent, Santa Clara, USA). Separation was conducted on an Agilent HP-5MS capillary column (30 m × 0.25 mm) with a constant helium flow rate of 1 mL/min. The temperature program was as follows: 40 °C maintained for 2 min, followed by a temperature increase of 10 °C/min to 250 °C, and it was held at 250 °C for 5 min. The mass spectrometry conditions were set as follows: interface temperature at 280 °C, ion source temperature at 230 °C, electron energy at 70 eV, and mass scan range from 40 to 550 amu. The reaction products were identified by comparing the retention times and mass spectra with authentic standards or by retrieving standard mass spectra in the NIST14 library.

### 4.7. Real-Time PCR

Total RNA was extracted from different tissues using a modified CTAB method, as previously described [37]. RNA extractions from petals at different flower developmental stages were carried out utilizing the Trizol method according to the manufacturer’s introduction (TaKaRa, Dalian, China). The first-strand cDNA was synthesized using the PrimerScript Reverse Kit (TaKaRa, Dalian, China). The real-time PCR primer pair for *HcJMT1* was designed using Primer Premier 5 (Table S1), and its amplification specificity was confirmed via agarose gel electrophoresis and melting curve analysis. The amplification efficiency and correlation coefficient of the primer were determined using the standard curve method with a dilution series of cDNA templates. Real-time PCR was performed on an ABI 7500 Real-Time PCR System (Applied Biosystems, Carlsbad, CA, USA) using SYBR Premix Ex Taq (TaKaRa, Dalian, China), as described previously [37,38]. Three independent amplifications were performed for each sample. Previously validated *HcACT* and *HcRPS* genes were used as reference genes for samples in different tissues and petals at different developmental stages, respectively [37]. The relative expression of *HcJMT1* was calculated using the 2^−ΔΔCt^ method [52]. Analysis of variance was conducted using SPSS 22 software with Tukey’s test (*p* = 0.05).

### 4.8. Headspace Collection and GC-MS Analysis of Floral MeJA

The emission of MeJA was analyzed using headspace collection and GC-MS method, as described previously [38]. Briefly, the entire flower at each stage was enclosed in a 500-mL glass bottle with the addition of 1.728 μg of ethyl caprate as the internal standard. After the equilibrium of volatiles and internal standard for 30 min, an SPME fiber (Supelco, Bellefonte, PA, USA) was inserted into the bottle to trap volatile compounds for 30 min. Then, trapped floral volatiles were analyzed using a GC-MS system, as described above. The MeJA isoforms were identified by comparing the retention times and mass spectra with the authentic standard. Quantification was calculated based on the peak area ratio and the quantity of the internal standard using the Agilent ChemStation Data Analysis Application. Analysis of variance was carried out via SPSS 22 software using Tukey’s test (*p* = 0.05).

## 5. Conclusions

Overall, we identified 12 SABATH family genes in the genome of *H. coronarium*. The gene structure, chromosome distribution, and phylogenetic analysis showed that tandem gene duplication is an important reason for the expansion of the SABATH family in *H. coronarium*, and this expansion occurred during the process of speciation after the separation of the Zingiberales and Poales lineages. Via phylogenetic analysis and expression profiling of HcSABATH members, *HcJMT1* was identified as the top candidate gene for floral MeJA biosynthesis. Functional characterization in vitro indicated that HcJMT1 is a jasmonic acid methyltransferase. The gene expression analysis and emission of floral MeJA suggested that *HcJMT1* is involved in the formation of floral MeJA in *H. coronarium*. Our results lay the basis for exploring the function of SABATH family members in *H. coronarium* and provide useful information for elucidating the molecular mechanism of floral MeJA biosynthesis in *H. coronarium*.

## Figures and Tables

**Table 1:** Summary of the members of the SABATH family in *Hedychium coronarium*.

Name	ID	Amino Acids Length	Molecular Weight (Da)	pI	Subfamily
HcBSMT1/HcSABATH1	novel01829	382	43,017.53	6.11	Clade VII
HcBSMT2/HcSABATH2	scaf_368.32	377	43,032.38	5.46	Clade VII
HcSABATH3	scaf_68.69	378	42,554.68	5.65	Clade VII
HcSABATH4	scaf_368.54	377	41,940.34	6.04	Clade VII
HcSABATH5	scaf_368.25	379	38,197.02	5.40	Clade VII
HcSABATH6	scaf_102.154.1	376	42,220.80	5.06	Clade VII
HcSABATH7	scaf_102.154.23	378	42,919.37	5.63	Clade VII
HcSABATH8	scaf_102.155.1	373	42,418.53	5.70	Clade VII
HcSABATH9	scaf_102.155.2	366	41,399.01	5.08	Clade VII
HcSABATH10	scaf_462.93	387	43,220.25	4.96	Clade V
HcJMT1/HcSABATH11	scaf_72.107	376	41,686.44	5.42	Clade V
HcJMT2/HcSABATH12	novel01692	376	41,583.13	5.19	Clade V

**Table 2:** Enzyme activity of recombinant HcJMT1 with different substrates.

Substrates	Enzyme Activity *
Jasmonic acid	+
Dihydrojasmonic acid	+
Salicylic acid	−
Benzoic acid	−
3-Hydroxybenzoic acid	−
2,3-Dihydnoxybenzoic acid	−
Cinnamic acid	−
o-Anisic acid	−
o-Coumaric acid	−
Nicotinic acid	−

* “+” indicates HcJMT1 exhibits activity with the substrate; “−” indicates no activity.

## Data Availability

All data are displayed in the manuscript and Supplementary Materials.

## Supplementary Materials

The following supporting information can be downloaded at: https://www.mdpi.com/article/10.3390/plants13010008/s1, Table S1: Primers for gene clone, bacterial expression, and real-time PCR of *HcJMT1*.
