# Supplementary material for: Identification and Characterization of Jasmonic Acid Methyltransferase Involved in the Formation of Floral Methyl Jasmonate in Hedychium coronarium
Source: Plants (Basel). 2023 Dec 19;13(1):8. doi: 10.3390/plants13010008 (PMC10780636; doi:10.3390/plants13010008)
Supplement: Supplementary file 1 [file plants-13-00008-s001.zip › plants-2711359-supplementary.pdf]

Supplementary Materials

Table S1. Primers for gene clone, bacterial expression and real-time PCR of *HcJMT1*.

Purpose	Forward/reverse primer sequence (5'-3')
Gene clone	ATGGATCTCAAGAAATATTTTCAC
	TCACTTCCTCACCAGAGCAAT
Bacterial expression	*Sal*I-GTCGACCGATGGATCTCAAGAAATATTTTCAC
	*Not*I-GCGGCCGCTCACTTCCTCACCAGAGCAATC
Real-time PCR	TACGCCAGCAACTCTAAGAT
	AAATCCGCAACGCTCATCGT
